# Resources, support, and integration as potential barriers and facilitators to the implementation of blended therapy in the routine care of inpatients: a qualitative study

**DOI:** 10.3389/fpsyt.2024.1417784

**Published:** 2024-12-17

**Authors:** Nikita Gupta, Sophie Leuba, Erich Seifritz, Thomas Berger, Wolfram Kawohl

**Affiliations:** ^1^ Clienia Schlössli AG, Psychiatry and Psychotherapy, Oetwil am See, Switzerland; ^2^ Department of Adult Psychiatry and Psychotherapy, University Hospital of Psychiatry Zurich (PUK), University of Zurich, Zurich, Switzerland; ^3^ Department of Clinical Psychology and Psychotherapy, University of Bern, Bern, Switzerland; ^4^ Medical School, University of Nicosia, Nicosia, Cyprus

**Keywords:** blended therapy, inpatient, qualitative study, implementation, resources, support, integration, barrier

## Abstract

While research on blended therapy (BT), i.e. the combination of face-to-face and digital treatment, has grown rapidly, integrating BT into routine practice remains limited, especially in inpatient settings. This study seeks to investigate the potential barriers healthcare providers and patients are confronted with in implementing BT to inpatients. Here, a retrospective, explorative qualitative research design was employed to gain insights into the experiences of healthcare professionals and inpatients in a real-world clinical setting. Specifically, we utilized semi-structured interviews to explore three key aspects: time resources, organizational support, and integration. A total of 11 therapists and 6 patients were interviewed. To our knowledge, this is one of the first studies to examine the implementation of blended therapy in the routine care of inpatients. We found that therapists emphasized several barriers including overwhelming workloads with insufficient time allocated for the work with the digital tools, inadequate time adjustments, a lack of ongoing training, and the necessity for a well-defined concept and setting of how to implement blended therapy. Interestingly, fewer barriers were reported by patients, who viewed the e-mental health platform as a valuable addition to their standard therapy. They also judged guidance and integration by their therapists as satisfactory and appreciated the adaptability offered in managing their workload in a flexible setting.

## 1 Introduction

Blended therapy (BT) is defined as the use of online or digital interventions, such as internet- based programs or smartphone apps, in conjunction with standard face-to-face (f-2-f) therapy (1). The combination of digital interventions and face-to-face therapy can be implemented in various forms. For example, the digital intervention can take place completely before or after the face-to-face therapy, or digital modules and face-to-face sessions can be integrated alternating into the treatment (2). A growing body of literature shows that computer- or internet-based interventions have comparable outcomes as regular f-2-f therapy while offering greater accessibility (3–7). Furthermore, other studies show that digital interventions, in addition to f-2-f therapy, lead to better outcomes than f-2-f therapy alone, with evidence of efficacy in depressive outpatients (8), inpatients and in aftercare settings (9, 10). However, caution is needed when making such comparisons. Studies like the one conducted by Merzhvynskaet al. (11) has shown that in these RCTs, the f-2-f group often exhibits more prognostic risk factors (e.g., higher severity, unemployment) compared to the BT group. Despite many advantages, such as increased patient engagement in their treatment, more flexible work conditions, or less traveling time for the patients, the implementation of BT in routine care is still slow (1, 3, 12, 13). To better understand such phenomena, several studies investigated potential barriers and facilitators of this process in an outpatient setting. Many studies argue that BT offers greater work flexibility and allows therapists to gain time and efficacy in their daily practice (1, 13). However, several studies have also demonstrated that therapists spend more time providing internet-based cognitive behaviour therapy or BT than while working with f-2-f therapy alone (3, 12, 14, 15). This can even lead to higher direct costs due to longer treatment durations because of inadequate implementation (16). The requirement for the extra time may result from the time needed during the intake period of the platform, for example, due to getting familiarized with the content and functions of the platform (17, 18). Furthermore time constraints have been shown to be a reason for reluctance to add new procedures in routine care (3, 19), for high time-pressure (14) and unmanageable workload (20). This shows that the implementation of BT should be carefully designed and requires adequate time resources for the involved healthcare professionals, as it seems that BT can lead to more overall workload than standard f-2-f therapy. We expect such barriers to be strongly present in the implementation of BT in inpatients’ routine care, where the time resources of healthcare providers are already limited. In practice, both healthcare providers and patients have often busy schedules, with healthcare providers having to ensure multiple tasks and while patients are engaged in intensive therapeutic programs. Another frequent barrier to implementing BT is the lack of support from the organization, including financial or logistical support, as well as inadequate training for healthcare providers and staff. Effectively, health professionals often mention the absence of clear guidelines, protocols, equipment, and financial resources, a lack of management, leadership, as well as the need for a clearly embedded concept (3, 13, 15, 20–23). The authors claimed that strong leadership and effective procedures to monitor the implementation and maintenance of BT are pertinent factors to successfully implement BT in routine care. In the context of the organization it is also important to mention the role of training that should be provided before implementing BT. The lack of knowledge and the need for training in web-based therapy (3, 23, 24), the low confidence in delivering online interventions and the uncertainty about the role of healthcare providers (14, 17) have also been a recurrent concern in past studies. Another noteworthy factor is the integration of online tools into f-2-f therapy. This also includes the uncertainty about which specific contents should be provided on the platform and to what extent and how the online material should be integrated into face-to-face therapy. Findings in the past have shown that therapists expressed the possibility of delegating psychoeducation aspects (17, 25) or administrative tools to an online platform (18). Several studies on healthcare professionals’ perspectives showed therapists wished to keep process-related aspects in their f-2-f therapy and use the online tools rather for homework, psychoeducation, and diaries (3, 26–28). Another relevant point for the inpatient setting, is the interprofessional collaboration and exchange between the nursing staff and the psychiatrist and psychologists. A study of Toonders et al. (17) reported that the interprofessional work added more value to the whole treatment. These studies suggest that involving the nursing staff is highly feasible and could strongly help to implement and maintain BT in the routine care of inpatients. However, there are no clear guidelines and protocols to integrate BT in routine care with inpatients and what the role of the different professions should play. The aim of this study is to explore the specific barriers and facilitators within the unique context of inpatient psychiatric care, a setting that remains notably underrepresented in BT research. We believe that our retrospective qualitative investigation will offer valuable insights to help address recurring implementation challenges in both outpatient and inpatient settings in the future. The study is based on the implementation in the psychiatric hospital *Clienia Schlössli AG* in 2021, where BT was initiated as part of a pilot project with the e-mental health programme *Minddistrict^R^*. However, it was found that BT was not systematically utilized and after a certain period of time, there was a noticeable decrease in the usage of the digital therapy. This raised the question about the factors influencing the difficulties in implementation. To understand better the key elements that can either facilitate or hinder the implementation of BT, we proceeded in two steps. First, we conducted informal interviews with therapists at the psychiatric hospital who had previously used BT. During these sessions, open-ended questions were asked, allowing participants to freely discuss various topics. In the next step, we conducted an extensive literature review on BT, digital therapies in general, and research on the implementation of new methods. Based on this combined approach, we identified the following core themes: resources, available support and management, and the integration of BT within the hospital. As an additional question particularly relevant in the context of inpatient care, we investigated the attitudes of therapists and patients towards the integration of BT within the context of sequential BT. Sequential BT is defined as the use digital interventions before or after a f-2-f treatment (29). Thus, we aimed to explore if the use of digital intervention before and after the inpatient care should be further developed. Previous studies have shown that the utilization of online and digital therapy can help maintain treatment success and reduce relapses when used in the aftercare (30–34). To gain in-depth “insight” into the experiences of health professionals and patients, we designed a retrospective exploratory qualitative approach based on similar qualitative studies examining outpatient settings (20, 35, 36). To our knowledge, there are only a few studies investigating the integration of BT in inpatient’s routine care (37).

## 2 Methods

### 2.1 Study context

This study aims to explore and understand the potential barriers and facilitators to the implementation of BT in inpatient psychotherapy encountered by the health care providers and patients. To collect our data and to identify barriers and facilitators we used a qualitative design method to gain insight into the experiences of health professionals and patients. We based our study on the experiences of health professionals and patients from the psychiatric hospital *Clienia Schlössli AG*. In 2021, this psychiatric hospital conducted a pilot project to implement blended therapy in their routine care. For six months, two wards used the e-mental health platform *Minddistrict^R^* with their inpatients. After the pilot project, lasting six months, the platform was not used and implemented anymore by the therapists even though the platform was still available to them. In 2022, some therapists independently relaunched the e-mental health platform and established it in their daily practice. *Minddistrict^R^* was founded in 2008 in the Netherlands and offers a transdiagnostic catalogue divided into modules, self-help training, and diaries. It offers the possibility of adapting the program individually to each patient by assigning suitable treatment modules to them. The treatment modules comprise various categories, such as diaries through which patients can document their behaviour patterns, psychoeducational chapters, and therapeutic interventions like exposure training. Patients can track behaviour patterns and therapy progress in diaries or select certain treatment modules independently in the self-help catalogue. Some treatment modules can only be used with direct companionship with the therapist. Additionally, *Minddistrict^R^* offers different communication channels such as video call or chat functions, which allow the therapist to validate the progress of the patient or the patient to communicate about content or questions directly to the therapist.

### 2.2 Participants and recruitment

Different strategies were selected for the recruitment, depending on the time period the e-mental health platform was used. Among the 27 eligible participants in the therapist’s group, eight therapists (29.6%) were no longer working in the psychiatric hospital, and some of them had no available contact address. The remaining part of the therapist received a written invitation (via e-mail or text message). Part of the therapists denied participation or did not respond at all (n=8, 29.6%). 40.7% of the therapists (n=11) who had worked or were currently working with *Minddistrict^R^* agreed on participation. The recruitment of the patient’s group was conducted by email and by phone. A part of the patient group, who were still in the psychiatric hospital during the recruitment phase, were directly recruited for the study via their therapist and then contacted by the research associate. Among the 32 eligible participants in the patient’s group, 23 patients (71.8%) did not respond to the invitation to participate. 18.8% of the patients (n=6) agreed to take part in this study. There were no dropouts. The recruitment was done continuously from March 2023 till June 2023. Non-responders were contacted repeatedly. We considered the sample sufficient to gain a variety of different experiences. There were no exclusion criteria unless someone declined the participation.

### 2.3 Data collection

After reviewing previous literature and informal exchanges with clinical experts, a semi-structured in-depth interview was developed. The interview guide was self-developed based on the predefined themes. Participants were asked to reflect on both facilitating and hindering aspects within these areas and were given space to provide suggestions for changes or adjustments. There were two interview guides developed, one for the therapist’s perspective and one for the patient’s perspective. The interviews consisted of fifteen open-ended questions (four on resources, three on support, five on integration, and three on the inpatient setting) for the therapist group and fourteen open-ended questions (four on resources, three on support, four on integration and three on inpatient setting) for the patient group. Some patients were more comfortable in English, so the semi-structured interviews were prepared in German and English (see **Supplementary**). The interviews were conducted by two researchers. The first researcher was a physician and clinician practicing at the clinic where the study was conducted, and this work was part of her doctoral dissertation. The second researcher was a psychology student completing this project as part of her master’s thesis at the university. Coding and analysis were also carried out by these two. Neither researcher had prior experience working with BT in a clinical setting before this study, allowing them to approach the research with an unbiased perspective regarding the outcomes. The physician who conducted the interviews was not involved in the treatment of the patients who were interviewed. Some of the therapists interviewed were current colleagues, while others were no longer working at the same hospital and therefore were not known to the physician in her day-to-day work. The interviews were conducted between March 2023 and June 2023. Depending on participants’ preferences and availability the interviews were conducted either in person in the hospital (n= 8) or by phone (n=9). The interviews were recorded via digital audio recordings, and anonymity was ensured by using code numbers for the names in the written transcription of the interviews. On average, the interviews lasted between 20 and 40 minutes.

### 2.4 Data analyses

Based on the study of Schuster et al. (38) who used a similar methodology, the recorded interviews were transcribed and analysed using a thematic analysis method for qualitative content because it allows meaningful patterns or topics to emerge with great flexibility (39) and an “allocation of observed phenomena within existing concepts, while it preserves the transmissibility for new phenomena” (38). Applying an inductive,data-driven method to deductive theory-driven content is considered a hybrid approach (40) and may help to bridge the gap between science and clinical practice (41). The recorded interviews in German (n=16) were transcribed verbatim and then translated into English. One interview was directly conducted and transcribed in English. The contents of the English written transcripts were then discussed with a research associate to ensure a meaningful translation. Data extraction and analysis were conducted using the English transcripts. Initially, both researchers read the transcriptions repeatedly to gain an overall understanding of the participants’ experience. Then, written transcripts (28’609 words) were uploaded into the qualitative software MAXQDA 2022 to allow us to conduct the thematic analysis as described by Braun and Clark (39). The principal codes were created and then reviewed, discussed, and validated in collaboration with a research associate through the analytic process with the possibility to rename or re-allocate meaningful units. Discrepancies and inaccuracies were addressed and resolved via further discussion and reflection. The full-text analysis resulted in a total of 474 code passages. The subsequent procedure closely mirrored the approach taken in the study Schuster et al. (2018), where the units were grouped into subtopics and validated by revisiting the corresponding text passages (38). After validating the units and their allocation, the refined subtopics were grouped into four topics which included our predefined topics. In this step, the allocation of the subtopics to the main topics was actively discussed with a research associate to reach an agreement. In the last step, the category system was revised and validated by an experienced researcher. Some subtopics were slightly rephrased for better understanding. At last, there was an overall agreement that the selected subtopics were coherent and adequate. We used a consensus rating approach. This repeated exchange between both main researchers and the experienced supervising researcher ensured that the data remained consistent and reliable, enhancing the results’ overall rigor, transparency, and reproducibility.

### 2.5 Ethical issues

The study protocol was submitted to the Cantonal Ethics Committee of Zurich via Swissethics. The Committee confirmed that the Federal Act on Research Involving Human Beings (HRA, RS 810.30) and the Ordinance on Human Research with the exception of clinical trials (HRA, RS 810.301, Art. 6-23) do not apply to this study (ProjectID: 2022-02290) because it is not part of the “research on diseases” category. Based on their Declaration of Non-Competence and after obtaining informed consent from all participants, we decided to pursue the research.

## 3 Results

### 3.1 Characteristics of participants

The data was collected from eleven therapists and six patients who worked with the platform between 2021 and 2023. The therapists (8 women, 3 men), reported as T1-T11 in the results, were either psychologists (n = 6), psychiatrists (n = 3) or nurses (n = 2). Their age range was from 26 to 59 years old (mean = 36.4, SD = 10.0). Six therapists worked with the platform in 2021, four therapists were actively working with the platform in 2023. One therapist worked with the platform in both time periods. Six therapists worked on a ward for stress-related disorders in adults of all ages. Five therapists worked on another ward, with a specialized focus on the treatment of depression in an age group of above 50 years. Three of the therapists were in a managing position, and one therapist was working as an intern. The patient group included six patients (all men), reported as P1-P6. Their age range was 24 to 59 years old (mean = 41.7, SD = 12.6). Three of them used the platform during the time period in 2021, and three of the patients used the platform in 2023.

### 3.2 Thematic analysis

Based on the 474 code passages acquired in data analysis of the 17 interviews identified 46 subtopics in the therapist interviews (see **Supplementary Table 1** in **Supplementary**) and 28 subtopics in the patient interviews (see **Supplementary Table 2** in **Supplementary**). They could be grouped into four main topics according to our predefined topics: (1) time resources, (2) support and organization, (3) integration, (4) sequential BT. In the therapist interviews, seven subtopics were directly associated with other subtopics and could also be identified as sub-subtopics.

### 3.3 Results

#### 3.3.1 Therapists’ perspective

##### 3.3.1.1 Time resources

One of the most frequent units identified regarding time resources was the time adjustment. Six therapists (54.5%) argued that there should be adjustments in their time resources. In this context they suggested fixed scheduled time and shorter or fewer therapeutic f-2-f sessions. For nine of the eleven therapists (81.8%) the utilization of the e-mental health platform was frequently experienced as additional work and effort that required extra time. Two of the therapists (18.2%) argued about the importance of resources allocated especially in the initial stage of introducing a new therapy. They expressed the need to have time available to get to know the tool and understand the offerings. They perceived that there was insufficient time allocated for their training. For two therapists (18.2%), introducing and explaining the e-mental health platform and the treatment modules to the patients was the biggest effort. As one of them mentioned:

*Because you had to explain it, and at the beginning, it somehow didn’t work for many because they didn’t quite understand it. Then you had to explain it again, and then you had to explain the whole project. You had to get them to sign up to participate, etc., so that was what cost more time. [T1]*

In contrast, two therapists (18.2%) did not experience the e-mental health platform as an overload in their daily routine. However, for one therapist, this was because the platform was not sufficiently implemented in the ward to change their daily work. Two therapists (18.2%) explicitly stated that the platform allowed them to work more efficiently and thus save some time in their daily practice. This increase in efficacy was due to self-assessment questionnaires or exercises like relaxation exercises being directly provided to them through the platform. Three other therapists (27.3%) who used the platform in 2021 found it beneficial during the COVID-19 pandemic because it enabled patients in physical isolation to continue working with the platform and effectively manage their time resources.

##### 3.3.1.2 Support and organization

The most frequent barrier reported was a lack of centralization and an overall concept provided by the psychiatric hospital regarding the implementation of BT. All therapists except one declared (n=10, 90.9%) that the more centralized and normalized procedures would be, the more it would counteract the work overload. For example, one therapist mentioned the necessity of integrating it into the regular administrative process so that there would be no time loss during the therapy sessions. Five therapists (45.5%) underlined a lack of resources and four of them (36.4%) stated that they would be willing only to go back to implementing more BT if there was a clearer concept and if the required resources would be clarified and allocated to them.

*Like every project in the clinic, I only want to take it on if it is a clear assignment and if it is also clear where the assignment comes from. You see, we need resources for training and to bring everyone to speed up [in the project], and only then it becomes a priority. [T4]*

Eight therapists (72.7%) mentioned that they received initial training and were satisfied with it. Five therapists (45.5%) reported having satisfactory support with technical problems and that occurring problems were quickly resolved by that person. Furthermore, three therapists (27.3%) reported having an experienced person who was more profoundly familiar with the platform as a “superuser” in charge of the ward to be very beneficial. In that way, the therapists did not have to spend a lot of time figuring things out on their own and could ask the “superuser” right away when struggling with questions regarding the platform.

*We were pleased that [therapist’s name], who was part of the ward, that worked out really well. And we could always ask him right away. We didn’t have to spend a lot of time figuring things out on our own because he was like a “superuser”. [ … ] I think people always went straight to [therapist’s name] when they had questions. [T4]*

Although the initial training was viewed as very positive and sufficient, four therapists (36.4%) emphasized a lack of ongoing support and training. It appeared that afterwards, therapists were given the flexibility and freedom to implement the e-mental health platform into their daily routines as they saw fit. Two therapists (18.2%) mentioned the necessity for monitoring and continuous support to ensure proper implementation. Another therapist suggested introducing supervision to learn from each other’s experiences. Additionally, five therapists (45.5%) mentioned having a table of contents as overview of the different modules would be helpful. Six therapists (54.5%) found the platform self-explanatory, well-structured, and easy to use in terms of features. Due to this there were seldom any questions or confusion regarding the platform itself. On the other hand, two therapists expressed uncertainty about how, how much, and when to use the platform, primarily because of a lack of clear guidance about the utilization. One therapist was unsure about the overall functionality of the platform, and both therapists were unsure what to write in the feedback through the chat function to the patients at the end of each module.

##### 3.3.1.3 Integration

Regarding the integration, all therapists except one (n=10, 90.9%) thought that the online treatment modules were a beneficial extension of the existing therapy program. Three therapists (27.2%) argued that it can promote autonomy and self-efficacy, and four therapists (36.4%) believed that highly motivated or tech-savvy patients could benefit from it the most. Nine therapists (81.8%) concluded that some parts of their therapy program, mainly psychoeducational content, could be replaced by the platform, allowing them to focus more on therapeutic processes. Five therapists (45.5%) also mentioned that some group therapies, like skills training or mindfulness exercises, could be replaced by online treatment modules.

*I find it helpful with basic interventions or psychoeducational elements like sleep hygiene, where you don’t have to explain them in as much detail. On the other hand, it gives you more time in individual sessions to focus on the outcome that has been achieved rather than having to dedicate time to sleep hygiene. [ … ] You can concentrate more on therapeutic topics in individual sessions and less on psychoeducational instruction. [T6]*

Three therapists (27.2%) clearly stated that the use of the platform could not replace the standard f-2-f therapy or psychotherapeutic process. All therapists reported that they only punctually integrated the platform’s contents into their individual therapy sessions or briefly asked at the beginning of their sessions if the patient had any questions or doubts. Three therapists (27.2%) mentioned possible integration adjustments. One therapist suggested integrating the treatment modules more into the individual therapy sessions, whereas another suggested integrating it into the interprofessional work, e.g., the weekly interprofessional reports. Lastly, one therapist underlined the importance of integrating it actively and continuously into the therapy, rather than simply assigning modules to the patients and expecting the patient to work through the assignments fully independently.

*It is crucial that we cannot simply provide content to the patient and expect them to read and do it. [ … ] For example, if we are addressing sleep hygiene for sleep disorders, it is important to address it directly with the patient. We provide the content and then discuss it together afterward. What doesn’t work is giving the patient 20 modules at the beginning of therapy and expecting them to go through them independently. So, completely handling this over to the patients and leaning back is not possible. We need to work through it with the patient actively. [T10]*

Regarding the platform’s content, four therapists (36.4%) found that the content provided was too broad and overwhelming. In contrast two therapists (18.2%) were satisfied with the possibility of selecting from a wide range of content. Two therapists (18.2%) even considered the offer too limited for psychoeducation and would even suggest adapting and extending the contents of the platform, for example, with self-assessment questionnaires.

*I would find it very enriching if there were more self-assessment questionnaire, where we could send the module to the patient with a questionnaire for screening, which they could then fill out online and the evaluation could also be done online. [ … ] I would see that as very beneficial and timesaving for my work, where I could argue for myself, ah if I could save time for diagnostic activities, then I can use this time to engage more with which modules from Minddistrict would be suitable for the patient. [T8]*

Another challenge mentioned by five therapists (45.5%) was that the utilization of the platform would not be suitable for all patients. One therapist mentioned that it was more adequate for patients who were not challenged enough by the existing therapy program. Additionally, one therapist mentioned that the willingness of the patients to work with such treatment modules would be crucial as this may otherwise lead to tensions in the therapeutic alliance if it was forced upon the patient. Finally, one therapist reminded that the use of digital intervention could also be dangerous in certain cases and, therefore must be carefully and cautiously implemented, depending on the assigned patient.

*That can also be a danger. Some people are distracted by it. They consume things and then engage themselves less in what is truly important for [them in] the future. [T3]*

Regarding interprofessional work, four therapists (36.4%) mentioned an ongoing exchange between the nursing staff and the psychologists or psychiatrist. However, seven therapists (63.6%) stated that there was either no or very little interprofessional exchange. Regarding the lack of interprofessional exchange two therapists believed that it was primarily attributed to the lack of integration. One therapist stated that a greater distribution of the workload to the interprofessional setting could be beneficial as there would be different perspectives involved what kind of modules might be most suited for the patient. On the other hand, one therapist mentioned the content was too specific to be discussed in interprofessional discourse.

##### 3.3.1.4 Sequential blended therapy

Six therapists (54.5%) perceived, in general, that the use of BT was not suitable in an inpatient context, and one therapist expressed the need for an adaption of the modules specifically to the situation of the inpatient context. Furthermore, five therapists (45.5%) found the use of treatment modules to be more meaningful in an outpatient setting, as they expressed that they were more fruitful in using the platform with outpatients. Three therapists (27.3%) expressed a positive outlook on patients accessing such a platform before entering a hospital. This might help bridge the waiting period or provide patients with content to read beforehand. This content could be to prepare them for their stay, explaining to them the daily schedule, the concept of psychotherapy, information regarding the condition, or the specific therapy form that is used in their future ward. On the other hand, five other therapists (45.5%) stated that it does not make sense to provide such a platform without any knowledge of the patients’ condition and symptoms. Additionally, two therapists (18.2%) mentioned financial and resource issues need to be allocated. Most therapists (n=8, 72.7%) viewed it positively that patients could continue using the platform after their stay. They proposed that this might be helpful for the patients to complete the topics they were working on during their stay and integrate them into their everyday lives. This might help maintain the effect of therapy, however therapists again highlighted resource issues in this context. This included concerns about who would be responsible for handling the platform after discharge and willingness to take over the BT of the therapist handling the patient after discharge. Moreover, two therapists (18.2%) also argued that it is beneficial for treatment, especially the therapeutic relationship, to have a conclusive ending with the discharge and the f-2-f sessions.

*We know this is a dangerous time [time after discharge], and sometimes the patients have to wait [for the next therapy session], and then they have something that offers them additional support. It is a very sensitive phase, where it is good to have something, even if it is, in quotation marks, “only” online interventions. [T11]*

#### 3.3.2 Patients’ perspective

##### 3.3.2.1 Time resources

Only one patient scheduled specific time to work on the assigned treatment modules. Three patients (50%) explicitly mentioned that the work did not feel overwhelming because they had enough free time to dedicate to the treatment modules. Three patients (50%) emphasized that this was related to the fact that there was no obligation to use the platform. Only one patient felt pressured to stay “up-to-date” with the treatment modules and integrate them into his daily routine. One patient highlighted that it could be stressful if using the platform was part of the routine practice. Additionally, two patients (33.3%) argued that adjustments should be made to their time resources, such as having a fixed schedule for working on the treatment modules, especially if its utilization becomes an integral part of the routine practice.

*If the idea is that I should work on it every day, then we should really have a time slot where we can work on it. Specifically, it should be indicated in our schedule. So that we can plan it. But if it’s just 1-2 times a week, then it’s fine as it is now. Then there is no need for integration [into our therapy plan]. [P6]*

##### 3.3.2.2 Support and organization

The patient’s primary support in using the platform was the therapist who assigned it to them. Four patients (66.7%) stated that this support was sufficient and did not wish for additional guidance. Only one patient expressed that he still felt uncertainty about how often they should work on the treatment modules and was missing guidelines of recommendation about the frequency of utilization.

##### 3.3.2.3 Integration

Regarding integration, all patients viewed the treatment modules as a valuable supplement to their therapy program. They found that the main benefit was gaining knowledge through new perspectives or repetition.

*I repeated everything again with the program after therapy. And because of that, it made me think about it again. And it has become clearer to me because I have written things down myself. [P5]*

Only one patient, who could not participate in certain group therapies because of a language barrier, stated that the use of the treatment modules was an actual replacement for those therapies. The rest of the patients clearly stated that the use of the modules could not replace traditional f-2-f therapy or psychotherapeutic processes.

From the patients’ perspective, five patients (83.3%) declared that the contents of the treatment modules were only punctually or very briefly integrated or discussed in their individual therapy sessions. For some of them, this was mainly because they had no questions, and the modules themselves were self-explanatory. Only one patient reported that his work with the platform was regularly checked and referred to during therapy sessions apart from when having questions. Possible integration adjustments were mentioned by three patients (50%). Two patients (33.3%) suggested that the platform’s contents could be more embedded or adjusted to the rest of the therapy program, like in the individual or group sessions. Regarding the contents, four patients (66.7%) underlined that it fit and were very specific to their existing therapy programs. While the majority of patients were satisfied with the contents of the platform (n=5, 83.3%), one patient found some exercises “weird” or unusual, and one patient, who already had extensive knowledge about his disorder from prior hospitalizations,therefore found the content to be nothing new. Regarding the use of the platform itself, five patients (83.3%) found it clear and easy to use in terms of its features. However, one patient expressed the desire to have more independence in working with the treatment modules without waiting for the therapist to unlock the access to the next modules. Four patients (66.7%) expressed positive views towards online tools, with one seeing them as an opportunity to save notes.

##### 3.3.2.4 Sequential blended therapy

One patient enjoyed not having his cell phone with him all the time and would recommend having available extra devices to work on the platform. The patient expressed concerns that spending additional time on his phone and possible distractions on it could lead to the deterioration of his mental state. Two patients declared that they would find it strange to receive modules to complete before their stay. One patient expressed the desire to have more information about his own symptoms before entering the psychiatric hospital. This patient also highlighted the potential benefit of filling out a questionnaire before entering the hospital to better capture their situation beforehand. This patient reported feeling significantly relieved upon entering the hospital and receiving support. This likely led to a distortion in the responses provided on the questionnaires, as he may have rated them more positively as compared to when filling them out at home. Two patients (33.3%) expressed the desire to continue using the platform after their stay.

## 4 Discussion

### 4.1 Time resources

Time resources were reported differently by patients and therapists. In our study, the patients declared using the platform did not lead to any time pressure as it was not perceived as integral part of the routine therapy program. In this context, some patients mentioned that if it becomes part of the routine practice, time adjustments such as fixed scheduled times when they should work on the platform should be implemented. Only one patient mentioned that the work on the treatment modules could cause stress. This concern has already been reported by healthcare providers who fear an increase in the patient’s burden and stress in their everyday lives (22, 25) and by patients who feel pressure and stress to complete their online tasks (38, 42). In general, it seems that in inpatient settings, where patients are relieved of everyday tasks, time constraints are less of a concern. At the same time, this must be balanced with the understanding that inpatients often have higher illness severity and lower levels of overall functioning. Additionally, the appreciation for the freedom when and how to work on the online modules reported by the patients in our study has been similarly reported in a previous study by Wilhelmsen et al. (43). In contrast, the issue of time resources was the second most frequent barrier reported by the therapists. For most of the therapists, the implementation of BT in their routine care was an additional work to their daily routine which was sometimes experienced as an overload. Some therapists underlined that extra effort and time were needed at the beginning to introduce and explain the platform to the patients and to familiarize oneself with the platform’s contents and functions. This underlines the necessity to allocate sufficient time resources at the beginning. Additionally, therapists in our study reported that the time spent on the treatment modules was not considered as therapy time even though they were spending more time than usual with the patient, e.g., introducing to the platform, brief contacts to inquire patients about their progress or support them when questions arose in between the therapy session. In this sense, we believe that therapists in our study probably used it as an add-on tool when they had the time and tended to put it aside when time resources were limited. To counteract this time overload, half of the therapists outlined the need for time adjustments such as fixed time specifically scheduled to work on the treatment modules, shorter therapeutic sessions, or fewer sessions to efficiently implement BT in their routine care. Several studies have shown that there is no compromise in the efficacy of using BT even with reduced f-2-f therapy time (27, 44–46), which indicates that also in this setting, an adjustment of the f-2-f time could be made without diminishing the therapy’s success. When considering the discussed aspects collectively, it becomes evident that careful planning is necessary for the implementation of BT, particularly regarding the management of time resources. While patients in this study generally appreciated the flexibility of BT, therapists perceived this approach as an additional burden. This was primarily because BT was not consistently recognized as part of formal therapy, and there was often no structured onboarding process for patients, leading to extra time investment by therapists. Hospitals could address these challenges by exploring concrete measures, such as integrating BT introduction into group therapy sessions, thereby streamlining the onboarding process and optimizing resource use. As long as therapists perceive BT as an additional time burden, it could become a significant barrier to successful implementation. Therefore, it is crucial to either demonstrate a clear benefit that outweighs the perceived effort or to find ways to reduce this perception of increased workload for therapists.

### 4.2 Support and organization

From the therapist’s perspective, the most frequent barrier reported was a lack of centralization or a clear concept of BT in the hospital. As reported by some therapists, the implementation of BT requires clear guidelines and stronger leadership, which should be provided by the organization. Additionally, some therapists also expressed feeling unsure about the platform’s contents and uncertainty about which module to refer to for which patient. So they wished for a better overview of the modules. Two therapists reported not knowing what to write in the feedback through the chat function, which was similarly reported by the therapists trained to use *Minddistrict^R^* in the Netherlands (18). Guidelines could serve to outline which modules are available on the platform, specify which modules are suitable for different patient groups (e.g., based on diagnosis, symptoms, or severity), and provide direction on how therapists should offer appropriate support throughout the process. Additionally, such guidelines could also support therapists by providing recommendations on usage frequency and offering guidance on writing effective feedback to patients. In this sense, BT should be an integral part of the organization with centralized and normalized procedures rather than a “nice to have” tool to maintain the implementation of BT. From the patient’s perspective, it was clearly stated that the use of the platform for them was not part of the routine practice but rather an additional offer they could use. This could explain why the need for a clearer blended concept was not an issue for them. Regarding the training and support, patients reported receiving sufficient guidance from their responsible therapist. Similarly, the therapists were highly satisfied to have a contact person to turn to in case of technical support. As the lack of technical support may be a recurrent factor leading to frustration and demotivation to use online tools (14, 28, 36), this was not the case in this study. Here therapists underlined the necessity of having “easy-going and super-accessible” contact persons, described as “super-user”. Most therapists were satisfied with the initial training, but they outlined a lack of expertise regarding the integration and the absence of supervision after their initial training. This finding is in line with previous studies reporting that ongoing consultations after the initial training play a critical role in the therapist’s adherence and skills regarding a new therapy form (18, 47). In summary, while some minor adjustments and additions to support mechanisms would have been desirable, many positive and helpful aspects were already in place. However, it is important to note that, similar to the issue of time management, therapists expressed a need for greater clarity and structure in this context as well.

### 4.3 Integration

All therapists and all patients considered the use of the platform as a beneficial addition to their standard therapy programs. The therapists argued that it brought new perspectives and useful repetitions for the patients and promoted the patients’ independence and self-efficacy. This finding is in line with previous findings in the literature, which showed betterment of self-efficacy and even of the therapeutic relationship (17, 18, 26, 48). Also, access to the patient’s written content is seen as a rich source of information, as similarly reported in previous studies (18, 38). In addition, many therapists reported that psychoeducational aspects could be replaced by the digital tool, allowing them to focus more on therapeutic processes in the individual sessions or adding self-assessment questionnaires to the platform could help save time in their daily routine. However, in general the platform seemed to be not entirely integrated into the f-2-f sessions but rather punctually checked up which led to experiencing it as a separate work. The therapists seemed reluctant to provide therapeutic interventions over the platform, reflecting health professionals’ persistent reluctance to delegate a large portion of the therapy process to online tools (3, 13, 26, 49). As another factor influencing adequate integration in the inpatient setting, there seemed to be little interprofessional exchange. One therapist stated that the work with the platform’s contents was too therapeutic to be exchanged in interprofessional discussion, while several therapists reported that a broader distribution of the tasks, including to the nursing staff, would be beneficial in terms of resources. Such findings underline the lack of a clear interprofessional organization and raise the question of how to involve the nursing staff in the implementation of BT in a beneficial way. This is especially relevant to clarify in the inpatient setting as the interprofessional collaboration and exchange between professionals play a fundamental role. Furthermore, the role of the nursing staff is highly relevant in the therapeutic process with the patient, and neglecting their roles in the context of blended therapy would result in omitting a significant portion. Overall, both therapists and patients viewed BT as a valuable addition to the treatment process. However, there remains uncertainty about how the digital tools should be effectively integrated with f-2-f therapy. This includes questions about the specific content of digital therapy as well as the involvement of the interprofessional team. Opinions among therapists diverged regarding the integration of BT into f-2-f sessions. Some therapists expressed a preference for greater incorporation of digital content into individual sessions, while others reported only using it selectively. This variation in practice highlights the differing approaches taken by therapists and has led to varying perspectives on the matter. Thus, the question of how BT should be integrated remains open, even after this study. It also remains uncertain whether the integration process should be standardized or whether therapists should be given the flexibility to tailor the use of BT based on their own preferences and the needs of their patients.

### 4.4 Sequential blended therapy

For half of the therapists, BT is not necessary or adequately adapted to an inpatient setting, and two of them explicitly stated that it would be more meaningful with outpatients where resources are more limited, while no patient expressed such concern. The possibility of having access to such a platform before entering the hospital presented mixed views in the therapists’ group. Some therapist considered it to be beneficial to have the possibility of providing different kinds of information before their admission, like about their stay, their condition, or the therapy program held in the ward. This underlines the therapist’s wish to broaden the functionalities of online tools (18). However, some therapists argued that it would not be useful to assign modules without knowing the patients, which was also reported by participants of the patients’ group. Most therapists thought it would be good to offer BT after the clinical stay, mainly to bridge the gap between the discharge and the aftercare as a critical and sensitive phase. This was also expressed by the patients, as they wished to maintain the effect of the inward therapy and wished to use the platform to integrate their learning into their everyday lives. However, a recurrent concern regarding the sequential BT from the therapists’ side was the lack of resources in the current situation to provide such an offer. Here, too, differing opinions emerged, suggesting that there is no single “right” way to treat a patient. Instead, it may be more appropriate to allow therapists and patients the flexibility to decide on the best approach. Sequential BT could be a valuable, individually tailored option for patient care. However, the critical issue of resource allocation must be addressed in advance, as this appears to be a highly relevant concern for therapists.

### 4.5 Limitations

This study has several noteworthy limitations. In the first place, it should be noted that the sample of our study consists of only six patients and eleven therapists. This means that our findings represent only a partial perspective on the full range of therapists and patients. Particularly the number of participating patients, is relatively small. Consequently, it is possible that full data saturation was not achieved, and our results may not fully represent the broader population. Furthermore, the study builds upon a voluntary self-selected group of participants, which may lead to a selection bias. A negative selection might have occurred due to participants with very negative experiences with the intervention not responding to the participation request in the first place. This type of selection bias could limit the generalizability of our findings, as it may create a skewed representation. Moreover, especially the therapists working with the e-mental Health platform in 2023 were doing this independently and without any outward motivation. Hence, there may already be a positive outlook and attitude towards BT in comparison to the general group of health care workers in routine care. Consequently, the absence of participants with strongly negative perspectives could mean that critical challenges or limitations of the intervention are underrepresented in the results, potentially providing an overly optimistic view of its implementation and impact. Additionally, there may also be a distortion in the given answers due to the two roles of one of the interviewers. One role as a researcher and another role as a therapist also working in the same hospital (but not with BT), and due to this, already known by part of the participants of the therapist’s group. This can make the participants more hesitant to share negative feedback openly. On the other hand, the familiarity can also work as a strength, leading to the participants being more honest and open with negative feedback. To prevent this kind of misrepresentation, the participants were encouraged in beforehand of every interview to state their opinions as freely as possible and the importance of positive as well as critical feedback for the further development of BT. Furthermore, part of the assessments was done two years after the e-mental health platform was used. The timely very distant memories can lead to distorted and less differentiated memories in contrast to the ones who used the platform very recently or at the time of the interview. An additional point to consider is that most participants in the therapist group were female, while all participants in the patient group were male. This gender imbalance may also limit the generalizability of our findings. A more balanced analysis could have been achieved by including more male participants in the therapist group and more female participants in the patient group, as they may have offered differing perspectives and insights.

### 4.6 Conclusion

This qualitative study has shown multiple possible barriers to the implementation of BT in the routine care of inpatients. As frequent barriers, the therapist mentioned work overload with insufficient time adjustments and missing ongoing training to efficiently maintain the use of BT in the long run. A key finding of this study underscores the significance of a well-defined concept and setting provided by hospital for successful implementation. A clear concept is essential in elucidating the role of BT. From the patient’s point of view, different facilitators to the implementation were reported, such as a satisfactory guidance and integration by their therapists and adequate workload given through flexible setting. In this paper, it is important to note that while we have described the different thematic areas separately, there is, of course, a close relationship between these areas and how they influence one another. For example, effective integration, interprofessional collaboration, and the simplification and centralization of certain processes could significantly reduce the additional workload reported by therapists in the “resources” category. Moreover, it should be emphasized that there were diverse opinions across the thematic areas, particularly among the group of therapists. Some therapists expressed a desire for clearer structures, while others preferred greater integration, with some finding the current level of integration sufficient. Additionally, there were differing preferences regarding the selection of modules: some therapists wished for more options, while others preferred to choose from a pre-selected set. This highlights the challenges faced by psychiatric hospitals in accommodating various needs and finding a balance that creates optimal conditions for all parties involved. Regarding the sequential form of BT, there were also mixed views. Therapists especially showed scepticism in the implementation of the sequential BT regarding the missing resources in proving guidance to patients which were not in the hospital. Taken together, the results show more barriers reported by the therapists in contrast to the patient’s perception, which underlines previous findings that understanding the patient’s perspective is crucial. However, healthcare providers may play the most essential role in the implementation and maintenance of BT.

## Data Availability

The raw data supporting the conclusions of this article will be made available by the authors, without undue reservation.

## References

[1] Schuster R Pokorny R Berger T Topooco N Laireiter A-R. The advantages and disadvantages of online and blended therapy: survey study amongst licensed psychotherapists in Austria. J Med Internet Res. (2018) 20:e11007. https://www.jmir.org/2018/12/e11007/ (Accessed August 2023). 30563817 10.2196/11007 PMC6315274

[2] Bielinski LL Trimpop L Berger T. Die Mischung macht’s eben? Blended-Psychotherapie als Ansatz der Digitalisierung in der Psychotherapie. Psychotherapeut. (2021) 66:447–54. doi: 10.1007/s00278-021-00524-3 PMC8268619 34257478

[3] Davies F Shepherd HL Beatty L Clark B Butow P Shaw J. Implementing web-based therapy in routine mental health care: Systematic review of health professionals’ perspectives. J Med Internet Res. (2020) 22. doi: 10.2196/17362 PMC7413287 32706713

[4] Andersson G Cuijpers P Carlbring P Riper H Hedman E. Guided Internet-based vs. face-to-face cognitive behavior therapy for psychiatric and somatic disorders: A systematic review and meta-analysis. World Psychiatry. (2014) 13:288–95. doi: 10.1159/000345967 PMC4219070 25273302

[5] Richards D Richardson T. Computer-based psychological treatments for depression: A systematic review and meta-analysis. Clin Psychol Rev. (2012) 32:329–342. doi: 10.1016/j.cpr.2012.02.004 22466510

[6] Ferrao Nunes-Zlotkowski K Shepherd HL Beatty L Butow P Shaw JM. Blended psychological therapy for the treatment of psychological disorders in adult patients: systematic review and meta-analysis. Interact J Med Res. (2024) 13:e49660. https://www.i-jmr.org/2024/1/e49660 (Accessed August 2023). 39470720 10.2196/49660 PMC11558224

[7] Mathiasen K Andersen TE Lichtenstein MB Ehlers LH Riper H Kleiboer A. The clinical effectiveness of blended cognitive behavioral therapy compared with face-to-face cognitive behavioral therapy for adult depression: randomized controlled noninferiority trial. J Med Internet Res. (2022) 24. doi: 10.2196/36577 PMC9543221 36069798

[8] Berger T Krieger T Sude K Meyer B Maercker A. Evaluating an e-mental health program (“deprexis”) as adjunctive treatment tool in psychotherapy for depression: Results of a pragmatic randomized controlled trial. J Affect Disord. (2018) 227:455–462. doi: 10.1016/j.jad.2017.11.021 29154168

[9] Zwerenz R Becker J Knickenberg RJ Siepmann M Hagen K Beutel ME. Online self-help as an add-on to inpatient psychotherapy: efficacy of a new blended treatment approach. Psychother Psychosom. (2017) 86:341–50. doi: 10.1159/000481177 29131090

[10] Diel A Schröter IC Frewer A-L Jansen C Robitzsch A Gradl-Dietsch G. A systematic review and meta analysis on digital mental health interventions in inpatient settings. NPJ Digit Med. (2024) 7:253. doi: 10.1038/s41746-024-01252-z 39289463 PMC11408664

[11] Merzhvynska M Wolf M Krieger T Berger T Munder T Watzke B. Prognostic risk factors in randomized clinical trials of face-to-face and internet-based psychotherapy for depression: A systematic review and meta-regression analysis. JAMA Psychiatry. (2024) 81:97–100. doi: 10.1001/jamapsychiatry.2023.3861 PMC10568439 37819635

[12] Hadjistavropoulos HD Alberts NM Nugent M Marchildon G. Improving access to psychological services through therapist-assisted, internet-delivered cognitive behaviour therapy. Can Psychol. (2014) 55:303–11. doi: 10.1037/a0037716

[13] Sander J Bolinski F Diekmann S Gaebel W Günther K Hauth I. Online therapy: an added value for inpatient routine care? Perspectives from mental health care professionals. Eur Arch Psychiatry Clin Neurosci. (2022) 272:107–18. doi: 10.1007/s00406-021-01251-1 PMC7961170 33725165

[14] Doukani A Free C Araya R Michelson D Cerga-Pashoja A Kakuma R. Practitioners’ experience of the working alliance in a blended cognitive–behavioural therapy intervention for depression: qualitative study of barriers and facilitators. BJPsych Open. (2022) 8. doi: 10.1192/bjo.2022.546 PMC9344874 35876079

[15] Titov N Dear B Nielssen O Staples L Hadjistavropoulos H Nugent M. ICBT in routine care: A descriptive analysis of successful clinics in five countries. Internet Interventions. (2018) 13:108–115. doi: 10.1016/j.invent.2018.07.006 PMC6112100 30206525

[16] Kenter RMF van de Ven PM Cuijpers P Koole G Niamat S Gerrits RS. Costs and effects of Internet cognitive behavioral treatment blended with face-to-face treatment: Results from a naturalistic study. Internet Interv. (2015) 2:77–83. doi: 10.1016/j.invent.2015.01.001

[17] Toonders SAJ Poolman EY Nieboer ME Pisters MF Veenhof C. Healthcare professionals’ perspectives on a blended care program in primary care; A qualitative study. Internet Interv. (2021) 26. doi: 10.1016/j.invent.2021.100440 PMC8358151 34401397

[18] Mol M van Genugten C Dozeman E van Schaik DJF Draisma S Riper H. Why uptake of blended internet-based interventions for depression is challenging: A qualitative study on therapists’ perspectives. J Clin Med. (2020) 9. doi: 10.3390/jcm9010091 PMC7019532 31905834

[19] Kivi M Eriksson MCM Hange D Petersson EL Björkelund C Johansson B. Experiences and attitudes of primary care therapists in the implementation and use of internet-based treatment in Swedish primary care settings. Internet Interv. (2015) 2:248–56. doi: 10.1016/j.invent.2015.06.001

[20] Wilhelmsen M Høifødt RS Kolstrup N Waterloo K Eisemann M Chenhall R. Norwegian general practitioners’perspectives on implementation of a guided web-based cognitive behavioral therapy for depression: A qualitative study. J Med Internet Res. (2014) 16. doi: 10.2196/jmir.3556 PMC4180343 25208886

[21] Vis C Mol M Kleiboer A Bührmann L Finch T Smit J. Improving Implementation of eMental Health for Mood Disorders in Routine Practice: Systematic Review of Barriers and Facilitating Factors. JMIR Ment Heal. (2018) 5:e20. http://mental.jmir.org/2018/1/e20/ (Accessed August 2023). 10.2196/mental.9769 PMC5878369 29549072

[22] Titzler I Berking M Schlicker S Riper H Ebert DD. Barriers and facilitators for referrals of primary care patients to blended internet-based psychotherapy for depression: Mixed methods study of general practitioners’ views. JMIR Ment Health. (2020) 7. doi: 10.2196/preprints.18642 PMC7463410 32673213

[23] Ross J Stevenson F Lau R Murray E. Factors that influence the implementation of e-health: A systematic review of systematic reviews (an update). Implementation Sci. (2016) 11. doi: 10.1186/s13012-016-0510-7 PMC5080780 27782832

[24] Folker AP Mathiasen K Lauridsen SM Stenderup E Dozeman E Folker MP. Implementing internet-delivered cognitive behavior therapy for common mental health disorders: A comparative case study of implementation challenges perceived by therapists and managers in five European internet services. Internet Interv. (2018) 11. doi: 10.1016/j.invent.2018.02.001 PMC6084870 30135761

[25] Bielinski LL Bur OT Wälchli G Suter JM Walsh N Kley MA. Two sides of the same coin? Patient and therapist experiences with a transdiagnostic blended intervention focusing on emotion regulation. Internet Interv. (2022) 30. doi: 10.1016/j.invent.2022.100586 PMC9663910 36386404

[26] Atik E Schückes M Apolinário-Hagen J. Patient and therapist expectations for a blended cognitive behavioral therapy program for depression: qualitative exploratory study. JMIR Ment Heal. (2022) 9. doi: 10.2196/preprints.36806 PMC9840101 36583934

[27] Kooistra LC Ruwaard J Wiersma JE van Oppen P van der Vaart R van Gemert-Pijnen JEWC. Development and initial evaluation of blended cognitive behavioural treatment for major depression in routine specialized mental health care. Internet Interv. (2016) 4. doi: 10.1016/j.invent.2016.01.003 PMC6096194 30135791

[28] Etzelmueller A Radkovsky A Hannig W Berking M Ebert DD. Patient’s experience with blended video- and internet based cognitive behavioural therapy service in routine care. Internet Interv. (2018) 12. doi: 10.1016/j.invent.2018.01.003 PMC6096318 30135780

[29] Erbe D Psych D Eichert HC Riper H Ebert DD. Blending face-to-face and internet-based interventions for the treatment of mental disorders in adults: Systematic review. J Med Internet Res. (2017) 19. doi: 10.2196/jmir.6588 PMC5622288 28916506

[30] Brakemeier EL Marchner J Gutgsell S Engel V Radtke M Tuschen-Caffier B. CBASP@home: Ein internetbasiertes situationsanalysen-training zur stabilisierung des therapieerfolgs nach stationärer therapie für chronisch depressive patienten. Verhaltenstherapie. (2013) 23. doi: 10.1159/000354814

[31] Ebert DD Hannig W Tarnowski T Sieland B Götzky B Berking M. Web-basierte Rehabilitationsnachsorge nach stationärer psychosomatischer Therapie (W-RENA). Rehabil. (2013) 52. doi: 10.1055/s-0033-1345191 23761205

[32] Ebert D Tarnowski T Gollwitzer M Sieland B Berking M. A transdiagnostic internet-based maintenance treatment enhances the stability of outcome after inpatient cognitive behavioral therapy: A randomized controlled trial. Psychother Psychosom. (2013) 82. doi: 10.1159/000345967 23736751

[33] Braun P Drüge M Hennemann S Nitsch FJ Staeck R Apolinário-Hagen J. Acceptance of E-mental health services for different application purposes among psychotherapists in clinical training in Germany and Switzerland: secondary analysis of a cross-sectional survey. Front Digit Heal. (2022) 4. doi: 10.3389/fdgth.2022.840869 PMC8918841 35295621

[34] Hennemann S Farnsteiner S Sander L. Internet- and mobile-based aftercare and relapse prevention in mental disorders: A systematic review and recommendations for future research. Internet Interventions. (2018) 14. doi: 10.1016/j.invent.2018.09.001 PMC6205252 30510909

[35] Schuster R Leitner I Carlbring P Laireiter AR. Exploring blended group interventions for depression: Randomised controlled feasibility study of a blended computer- and multimedia-supported psychoeducational group intervention for adults with depressive symptoms. Internet Interv. (2017) 8:63–71. doi: 10.1016/j.invent.2017.04.001 30135830 PMC6096250

[36] Titzler I Saruhanjan K Berking M Riper H Ebert DD. Barriers and facilitators for the implementation of blended psychotherapy for depression: A qualitative pilot study of therapists’ perspective. Internet Interv. (2018) 12:150–64. doi: 10.1016/j.invent.2018.01.002 PMC6096333 30135779

[37] Bielinski LL Wälchli G Nissen C Berger T Moggi F. Does an internet-based emotion regulation intervention provide added value for acute psychiatric inpatient care? Protocol for a randomized controlled pilot trial. JMIR Res Protoc. (2023) 12. doi: 10.2196/47656 PMC10369307 37432724

[38] Schuster R Sigl S Berger T Laireiter AR. Patients’ Experiences of web- And mobile-assisted group therapy for depression and implications of the group setting: Qualitative follow-up study. JMIR Ment Heal. (2018) 5. doi: 10.2196/mental.9613 PMC6060305 29997106

[39] Braun V Clarke V. Using thematic analysis in psychology,Qualitative Research in Psychology. J Chem Inf Model. (2008) 3. doi: 10.1191/1478088706qp063oa

[40] Fereday J Muir-Cochrane E. Demonstrating rigor using thematic analysis: A hybrid approach of inductive and deductive coding and theme development. Int J Qual Methods. (2006) 5. doi: 10.1177/160940690600500107

[41] Cochrane LJ Olson CA Murray S Dupuis M Tooman T Hayes S. Gaps between knowing and doing: Understanding and assessing the barriers to optimal health care. J Contin Educ Health Prof. (2007) 27:94–102. doi: 10.1002/chp.106 17576625

[42] Braun P Atik E Guthardt L Apolinário-Hagen J Schückes M. Barriers to and facilitators of a blended cognitive behavioral therapy program for depression and anxiety based on experiences of university students: qualitative interview study. JMIR Form Res. (2023) 7. doi: 10.2196/45970 PMC10134026 37043272

[43] Wilhelmsen M Lillevoll K Risør MB Høifødt R Johansen M-L Waterloo K. Motivation to persist with internet-based cognitive behavioural treatment using blended care: a qualitative study. BMC Psychiatry. (2013) 13:296. doi: 10.1186/1471-244X-13-296 24199672 PMC4226213

[44] Kenwright M Liness S Marks I. Reducing demands on clinicians by offering computer-aided self-help for phobia/panic. Feasibility study. Br J Psychiatry. (2001) 179. doi: 10.1192/bjp.179.5.456 11689405

[45] Sharry J Davidson R McLoughlin O Doherty G. A service-based evaluation of a therapist-supported online cognitive behavioral therapy program for depression. J Med Internet Res. (2013) 15. doi: 10.2196/jmir.2248 PMC3713925 23807565

[46] Wright JH Wright AS Albano AM Basco MR Goldsmith LJ Raffield T. Computer-assisted cognitive therapy for depression: Maintaining efficacy while reducing therapist time. Am J Psychiatry. (2005) 162(6):1158–64. doi: 10.1176/appi.ajp.162.6.1158 15930065

[47] Beidas RS Edmunds JM Marcus SC Kendall PC. Training and consultation to promote implementation of an empirically supported treatment: A randomized trial. Psychiatr Serv. (2012) 63. doi: 10.1176/appi.ps.201100401 PMC3432154 22549401

[48] Robertson L Smith M Castle D Tannenbaum D. Using the internet to enhance the treatment of depression. Australas Psychiatry. (2006) 14:413–7. doi: 10.1080/j.1440-1665.2006.02315.x 17116083

[49] Marks IM Kenwright M McDonough M Whittaker M Mataix-Cols D. Saving clinicians’ time by delegating routine aspects of therapy to a computer: A randomized controlled trial in phobia/panic disorder. Psychol Med. (2004) 34:9–17. doi: 10.1017/S003329170300878X 14971623

