# Supplementary material for: Resources, support, and integration as potential barriers and facilitators to the implementation of blended therapy in the routine care of inpatients: a qualitative study
Source: Front Psychiatry. 2024 Dec 17;15:1417784. doi: 10.3389/fpsyt.2024.1417784 (PMC11687372; doi:10.3389/fpsyt.2024.1417784)
Supplement: Supplementary file 1 [file DataSheet1.pdf]

Supplement

1. Interview guide in German

1.1 Questions for health care provider

Questions about resources

- Wie veränderte sich Ihre tägliche Stationsarbeit durch die Arbeit mit der e-Mental Health Plattform? Welchen Einfluss hatte dies auf Ihre zeitlichen Ressourcen?
- Fakultativ: Mit welchem Mitteln wurde einer allfälligen Mehrbelastung entgegengewirkt?
- Was von Ihrer vorbestehenden Arbeit könnte allenfalls ersetzt werden bzw. weglassen werden durch die Anwendung von Minddistrict?
- Welche Anpassungen könnten vorgenommen werden, damit es zu einer (zusätzlichen) Erleichterung Ihrer klinischen Tätigkeit kommt?

Questions about support

- Welche Unterstützung und Anleitungen haben Sie bei Ihrer Arbeit mit der e-Mental Health Plattform erhalten? Gab es interne Guidelines, Supervisionen oder Ansprechpartner auf der Station, wenn es zu Schwierigkeiten oder Fragen zu der Anwendung kam?
- Was war bei den vorhandenen Unterstützungsmöglichketen gut? Was hatte Ihnen gefehlt?
- Was würden Sie sich als zusätzliche Unterstützung in der Zukunft wünschen?

Questions about integration

- Wie wurden die Inhalte der e-Mental Health Plattform in die face-to-face Therapie eingebaut?
- Welche Anpassungen erfolgten bezüglich der Dauer der Einzelgespräche, der Inhalte oder der Anzahl Therapiesitzungen?
- Wie wurden mit den Inhalten der e-Mental Health Plattform umgegangen im Team? Wie erfolgte der interprofessionelle Austausch mit beispielsweise dem Pflegeperson/Therapeuten?
- Welche Aspekte der Integration in die Einzelgespräche und in das interprofessionelle Team haben gut funktioniert? Was hat Ihnen gefehlt?
- Auf was sollte in der Zukunft bei der Integration geachtet werden?

Closing questions

- Wie schätzen Sie im generellen das zukünftige Potential von Therapien mittels digitaler Hilfsmittel ein?
- Sehen Sie Möglichkeiten wie die e-Mental Health Plattform vor oder nach dem stationären Aufenthalt sinnvoll eingesetzt werden könnte?
- Würden Sie gerne (wieder) vermehrt die BT im stationären Rahmen einbauen?

1.2 Questions for patients

Questions about resources

- Wie veränderte sich Ihr Alltag auf der Station durch die Arbeit an der e-Mental Health Plattform neben dem bevorstehenden Therapieprogramm?
- Wann habe Sie an den Modulen gearbeitet und wie haben Sie dies in Ihren Alltag eingeplant? Wie hat das für Sie funktioniert?
- Welche Therapieelemente im Stationsprogramm könnten allenfalls aufgrund der Arbeit mit den Modulen ersetzt bzw. weggelassen werden könnten?
- Wie sollten die Module aus Ihrer Sicht idealerweise in das Therapieprogrammen bzw. in Ihren Alltag integriert werden?

Questions about support

- Welche Unterstützung und Anleitungen haben Sie bei Ihrer Arbeit mit den Modulen auf der e-Mental Health Plattform erhalten?

- Was war bei den vorhandenen Möglichkeiten gut? Was hat Ihnen gefehlt?
- Was würden Sie sich als zusätzliche Unterstützung in der Zukunft wünschen?

Questions about integration

- Inwiefern haben die Module der e-Mental Health Plattform in Ihr übriges Therapieprogramm gepasst?
- Wie wurden die Inhalte der e-Mental Health Plattform in die anderen Therapien, beispielsweise in den Einzel- und Gruppentherapien, eingebaut?
- Welche Aspekte der Integration haben gut funktioniert? Was hat Ihnen gefehlt?
- Auf was sollte in der Zukunft bei der Integration geachtet werden?

Closing questions

- Wie schätzen Sie generell das zukünftige Potential von Therapien mittels digitaler Hilfsmittel ein?
- Sehen Sie Möglichkeiten wie die e-Mental Health Plattform vor oder nach dem stationären Aufenthalt sinnvoll eingesetzt werden könnte?
- Falls es erneut zu einem stationären Eintritt kommen sollte, würde Sie gerne wieder diese e-Mental Health Plattform nutzen?

2. Interview guide in English

2.1 Questions for health care provider

Questions about resources

- How did your daily work at the clinical change with the addition of the e-mental health platform?
- What impact did this have on your time resources / time management?
- Facultative: What could counteract the additional load of work?
- What of your preexisting work could be replaced and thereby omitted by the usage of Minddistrict?
- What adjustments would be necessary to make to (additionally) facilitate your clinical work?

Questions about support

- What support and guidance have you received in your work with the e-mental health platform? Were there internal guidelines, supervisions, or contacts on the clinical ward when difficulties or questions arose in using the e-mental health platform?
- What was good about the support options available? What was missing?
- What would you like to have as additional support in the future?

Questions about integration

- How was the e-mental health platform content integrated into the face-to-face therapy with patients?
- What adjustments were successfully made in terms of the duration of individual sessions, its content, or the number of sessions?
- How was the content of the e-mental health platform handled in the team working together? How was the interprofessional exchange for example with the nurses/therapists?
- What aspects of the integration into the individual sessions and into the interprofessional team worked well? What was missing for you?
- What should be considered in the future regarding the integration?

Closing questions:

- How do you estimate the potential of using digital aid for future therapies?
- How could the e-mental health platform be meaningfully used before or after an inpatient's stay? What possibilities do you see?
- Would you like to incorporate more BT in the routine care of inpatients?

2.2 Questions for patients

Questions about resources

- How did your daily life at the clinical ward change with the work on the e-mental health platform modules alongside with the pre-existing therapy program?
- When did you work on the modules and how did you schedule this into your daily routine? How did it work for you?
- Which elements of the therapeutic program on the ward can be replaced by the work with the modules and which elements could thus be omitted?
- From your perspective, how should the modules ideally be integrated into the therapy program or into your daily life at the hospital?

Questions about support

- What support and guidance did you receive when working with the modules of the e-mental health platform?
- What was good about the support options available? What was missing for you?
- What would you like to have as additional support in the future?

Questions about integration

- To what extent did the modules of the e-mental health platform fit into the rest of your therapy program?
- How was the content of the e-mental health platform integrated into your other therapies, such as individual and group therapies?
- What aspects of the integration worked well? What was missing for you?
- What should be considered in the future regarding the integration?

Closing questions

- How do you estimate the potential of using digital aid for future therapies?
- How could the e-mental health platform be meaningfully used before or after an inpatient's stay? What possibilities do you see?
- If you were to be an inpatient at a hospital again, would you like to use this e-mental health platform again?

3. Tables result

Table 1
Therapist perspectives (category system)

Main themes<br>Subthemes	N therapists (%)
**Time resources**	
• Extra effort	9 (81.8)
• Introduction time	2 (18.2)
• Time adjustments needed	6 (54.5)
• No time overload	2 (18.2)
• Time saved	2 (18.2)
o Covid pandemic	3 (27.3)
**Support and organization**	
• Missing centralization or clear concept	10 (90.9)
• Lack of resources	5 (45.5)
• Sufficient initial training	8 (72.7)
• Good technical support with contact person	5 (45.5)
• Experienced person in charge (superuser)	3 (27.3)
• Overview of the platform contents	2 (18.2)
• Wish for overview and "which fits who" guideline	2 (18.2)
• Need for ongoing support, training, and monitoring	2 (18.2)
• Lack of knowledge of how to give feedback	2 (18.2)
• Interprofessional exchange existing	4 (36.4)
• No interprofessional exchanges existing	7 (63.6)
o Not implemented enough	2 (18.2)
o Contents too therapeutic	1 (9.1)
**Integration**	
• Good supplement	10 (90.9)
• Therapy replacement	9 (81.8)
o For psychoeducation	9 (81.8)
o For mindfulness exercises	3 (27.3)
o For skill training	2 (18.2)

• No therapy replacement	3 (27.3)
• Consumption warning	1 (9.1)
• Punctually or briefly integrated	11 (100)
• Possible integration adjustments	3 (27.3)
• Wish to select adapted contents	2 (18.2)
• Offer too broad	4 (36.4)
• Wish for broader offer	2 (18.2)
• Wish for inward-specific modules	1 (9.1)
• Expand to other functionalities	1 (9.1)
• Digitalization seen as inevitable	1 (9.1)
• Too new / too unknown	1 (9.1)
• Not adapted to all patients	5 (45.5)
**Sequential BT**	
• Not adapted or necessary for inward	6 (54.5)
• More meaningful or several advantages with outpatients	5 (45.5)
• Missing resources with outpatients	1 (9.1)
Before the stay in hospital	
• Access to platform before stay viewed as positive	3 (27.3)
• Idea to give patients information to prepare before admission	2 (18.2)
• Not recommendable to assign modules without knowledge of patient	5 (45.5)
After the stay in hospital	
• Positive to use after stay as transition	8 (72.7)
• Wanting a clear end the inward treatment with discharge	2 (18.2)

Table 2

Patient's perspectives (category system)

Main themes<br>Subthemes	N patients (%)
**Time resources**	
• Usage in free time	6 (100)
• Not scheduled when to use	5 (83.3)
• No time overload	3 (50)
• Not seen mandatory	3 (50)
• Time adjustments required if it should be mandatory	2 (33.3)
• Stressful if it should be mandatory	1 (16.7)
• Exiting pressure to be "up to date"	1 (16.7)
**Support and organization**	
• Support and guidance available by responsible therapist	3 (50)
• Satisfied with existing support and guidance	4 (66.7)
• Frequency of usage unclear	1 (16.7)
• Platform seen as clear, intuitive, and self-explanatory	5 (83.3)
**Integration**	
• Good as a supplement	6 (100)
• Therapy replacement possible	1 (16.7)
• No replacement of therapy possible	5 (83.3)
• Contents were not integrated	5 (83.3)
• Could be more embedded in f2f	2 (33.3)
• Viewed as part of therapy program (integrated)	1 (16.7)
• Contents fit to therapy	4 (66.7)
• Contents nothing new	1 (16.7)
• Contents viewed as not matching	1 (16.7)
• Different language version needed of some modules	1 (16.7)
• Wish to work more independently	1 (16.7)
• Positive outlook towards digitalization	4 (66.7)
• Importance to adopt right attitude for the use of BT	1 (16.7)

Sequential BT

• Wish not to use their own phone during stay / recommend extra device	1 (16.7)
Before the stay in hospital	
• Strange to receive module before admission	3 (27.3)
• Wish for more information on symptoms before admission	1 (16.7)
After the stay in hospital	
• Would continue using after discharge	2 (33.3)
