# Supplementary material for: Resources, support, and integration as potential barriers and facilitators to the implementation of blended therapy in the routine care of inpatients: a qualitative study
Source: Front Psychiatry. 2024 Dec 17;15:1417784. doi: 10.3389/fpsyt.2024.1417784 (PMC11687372; doi:10.3389/fpsyt.2024.1417784)
Supplement: Supplementary file 2 [file Table1.docx]

**Supplement**

1. **Interview guide in German**
   1. **Questions for health care provider**

Questions about resources

- Wie veränderte sich Ihre tägliche Stationsarbeit durch die Arbeit mit der e-Mental Health Plattform? Welchen Einfluss hatte dies auf Ihre zeitlichen Ressourcen?
- Fakultativ: Mit welchem Mitteln wurde einer allfälligen Mehrbelastung entgegengewirkt?
- Was von Ihrer vorbestehenden Arbeit könnte allenfalls ersetzt werden bzw. weglassen werden durch die Anwendung von Minddistrict?
- Welche Anpassungen könnten vorgenommen werden, damit es zu einer (zusätzlichen) Erleichterung Ihrer klinischen Tätigkeit kommt?

Questions about support

- Welche Unterstützung und Anleitungen haben Sie bei Ihrer Arbeit mit der e-Mental Health Plattform erhalten? Gab es interne Guidelines, Supervisionen oder Ansprechpartner auf der Station, wenn es zu Schwierigkeiten oder Fragen zu der Anwendung kam?
- Was war bei den vorhandenen Unterstützungsmöglichketen gut? Was hatte Ihnen gefehlt?
- Was würden Sie sich als zusätzliche Unterstützung in der Zukunft wünschen?

Questions about integration

- Wie wurden die Inhalte der e-Mental Health Plattform in die face-to-face Therapie eingebaut?
- Welche Anpassungen erfolgten bezüglich der Dauer der Einzelgespräche, der Inhalte oder der Anzahl Therapiesitzungen?
- Wie wurden mit den Inhalten der e-Mental Health Plattform umgegangen im Team? Wie erfolgte der interprofessionelle Austausch mit beispielsweise dem Pflegeperson/Therapeuten?
- Welche Aspekte der Integration in die Einzelgespräche und in das interprofessionelle Team haben gut funktioniert? Was hat Ihnen gefehlt?
- Auf was sollte in der Zukunft bei der Integration geachtet werden?

Closing questions

- Wie schätzen Sie im generellen das zukünftige Potential von Therapien mittels digitaler Hilfsmittel ein?
- Sehen Sie Möglichkeiten wie die e-Mental Health Plattform vor oder nach dem stationären Aufenthalt sinnvoll eingesetzt werden könnte?
- Würden Sie gerne (wieder) vermehrt die BT im stationären Rahmen einbauen?
  1. **Questions for patients**

Questions about resources

- Wie veränderte sich Ihr Alltag auf der Station durch die Arbeit an der e-Mental Health Plattform neben dem bevorstehenden Therapieprogramm?
- Wann habe Sie an den Modulen gearbeitet und wie haben Sie dies in Ihren Alltag eingeplant? Wie hat das für Sie funktioniert?
- Welche Therapieelemente im Stationsprogramm könnten allenfalls aufgrund der Arbeit mit den Modulen ersetzt bzw. weggelassen werden könnten?
- Wie sollten die Module aus Ihrer Sicht idealerweise in das Therapieprogrammen bzw. in Ihren Alltag integriert werden?

Questions about support

- Welche Unterstützung und Anleitungen haben Sie bei Ihrer Arbeit mit den Modulen auf der e-Mental Health Plattform erhalten?
- Was war bei den vorhandenen Möglichkeiten gut? Was hat Ihnen gefehlt?
- Was würden Sie sich als zusätzliche Unterstützung in der Zukunft wünschen?

Questions about integration

- Inwiefern haben die Module der e-Mental Health Plattform in Ihr übriges Therapieprogramm gepasst?
- Wie wurden die Inhalte der e-Mental Health Plattform in die anderen Therapien, beispielsweise in den Einzel- und Gruppentherapien, eingebaut?
- Welche Aspekte der Integration haben gut funktioniert? Was hat Ihnen gefehlt?
- Auf was sollte in der Zukunft bei der Integration geachtet werden?

Closing questions

- Wie schätzen Sie generell das zukünftige Potential von Therapien mittels digitaler Hilfsmittel ein?
- Sehen Sie Möglichkeiten wie die e-Mental Health Plattform vor oder nach dem stationären Aufenthalt sinnvoll eingesetzt werden könnte?
- Falls es erneut zu einem stationären Eintritt kommen sollte, würde Sie gerne wieder diese e-Mental Health Plattform nutzen?

1. **Interview guide in English**
   1. **Questions for health care provider**

Questions about resources

- How did your daily work at the clinical change with the addition of the e-mental health platform?
- What impact did this have on your time resources / time management?
- Facultative: What could counteract the additional load of work?
- What of your preexisting work could be replaced and thereby omitted by the usage of Minddistrict?
- What adjustments would be necessary to make to (additionally) facilitate your clinical work?

Questions about support

- What support and guidance have you received in your work with the e-mental health platform? Were there internal guidelines, supervisions, or contacts on the clinical ward when difficulties or questions arose in using the e-mental health platform?
- What was good about the support options available? What was missing?
- What would you like to have as additional support in the future?

Questions about integration

- How was the e-mental health platform content integrated into the face-to-face therapy with patients?
- What adjustments were successfully made in terms of the duration of individual sessions, its content, or the number of sessions?
- How was the content of the e-mental health platform handled in the team working together? How was the interprofessional exchange for example with the nurses/therapists?
- What aspects of the integration into the individual sessions and into the interprofessional team worked well? What was missing for you?
- What should be considered in the future regarding the integration?

Closing questions:

- How do you estimate the potential of using digital aid for future therapies?
- How could the e-mental health platform be meaningfully used before or after an inpatient’s stay? What possibilities do you see?
- Would you like to incorporate more BT in the routine care of inpatients?
  1. **Questions for patients**

Questions about resources

- How did your daily life at the clinical ward change with the work on the e-mental health platform modules alongside with the pre-existing therapy program?
- When did you work on the modules and how did you schedule this into your daily routine? How did it work for you?
- Which elements of the therapeutic program on the ward can be replaced by the work with the modules and which elements could thus be omitted?
- From your perspective, how should the modules ideally be integrated into the therapy program or into your daily life at the hospital?

Questions about support

- What support and guidance did you receive when working with the modules of the e-mental health platform?
- What was good about the support options available? What was missing for you?
- What would you like to have as additional support in the future?

Questions about integration

- To what extent did the modules of the e-mental health platform fit into the rest of your therapy program?
- How was the content of the e-mental health platform integrated into your other therapies, such as individual and group therapies?
- What aspects of the integration worked well? What was missing for you?
- What should be considered in the future regarding the integration?

Closing questions

- How do you estimate the potential of using digital aid for future therapies?
- How could the e-mental health platform be meaningfully used before or after an inpatient’s stay? What possibilities do you see?
- If you were to be an inpatient at a hospital again, would you like to use this e-mental health platform again?

1. **Tables result**

| **Table 1** | |
| --- | --- |
| Therapist perspectives (category system) | |
| Main themes | N therapists |
| Subthemes | (%) |
| **Time resources** |  |
| - Extra effort | 9 (81.8) |
| - Introduction time | 2 (18.2) |
| - Time adjustments needed | 6 (54.5) |
| - No time overload | 2 (18.2) |
| - Time saved   - Covid pandemic | 2 (18.2)  3 (27.3) |
| **Support and organization** |  |
| - Missing centralization or clear concept | 10 (90.9) |
| - Lack of resources | 5 (45.5) |
| - Sufficient initial training | 8 (72.7) |
| - Good technical support with contact person | 5 (45.5) |
| - Experienced person in charge (superuser) | 3 (27.3) |
| - Overview of the platform contents | 2 (18.2) |
| - Wish for overview and “which fits who” guideline | 2 (18.2) |
| - Need for ongoing support, training, and monitoring | 2 (18.2) |
| - Lack of knowledge of how to give feedback | 2 (18.2) |
| - Interprofessional exchange existing | 4 (36.4) |
| - No interprofessional exchanges existing   - Not implemented enough   - Contents too therapeutic | 7 (63.6)  2 (18.2)  1 (9.1) |
| **Integration** |  |
| - Good supplement | 10 (90.9) |
| - Therapy replacement   - For psychoeducation   - For mindfulness exercises   - For skill training | 9 (81.8) 9 (81.8) 3 (27.3) 2 (18.2) |
| - No therapy replacement | 3 (27.3) |
| - Consumption warning | 1 (9.1) |
| - Punctually or briefly integrated | 11 (100) |
| - Possible integration adjustments | 3 (27.3) |
| - Wish to select adapted contents | 2 (18.2) |
| - Offer too broad | 4 (36.4) |
| - Wish for broader offer | 2 (18.2) |
| - Wish for inward-specific modules | 1 (9.1) |
| - Expand to other functionalities | 1 (9.1) |
| - Digitalization seen as inevitable | 1 (9.1) |
| - Too new / too unknown | 1 (9.1) |
| - Not adapted to all patients | 5 (45.5) |
| **Sequential BT** |  |
| - Not adapted or necessary for inward | 6 (54.5) |
| - More meaningful or several advantages with outpatients | 5 (45.5) |
| - Missing resources with outpatients | 1 (9.1) |
| *Before the stay in hospital* |  |
| - Access to platform before stay viewed as positive | 3 (27.3) |
| - Idea to give patients information to prepare before admission | 2 (18.2) |
| - Not recommendable to assign modules without knowledge of patient | 5 (45.5) |
| *After the stay in hospital* |  |
| - Positive to use after stay as transition | 8 (72.7) |
| - Wanting a clear end the inward treatment with discharge | 2 (18.2) |

| **Table 2** |  |
| --- | --- |
| Patient’s perspectives (category system) |  |
| Main themes  Subthemes | N patients  (%) |
| **Time resources** |  |
| - Usage in free time | 6 (100) |
| - Not scheduled when to use | 5 (83.3) |
| - No time overload | 3 (50) |
| - Not seen mandatory | 3 (50) |
| - Time adjustments required if it should be mandatory | 2 (33.3) |
| - Stressful if it should be mandatory | 1 (16.7) |
| - Exiting pressure to be “up to date” | 1 (16.7) |
| **Support and organization** |  |
| - Support and guidance available by responsible therapist | 3 (50) |
| - Satisfied with existing support and guidance | 4 (66.7) |
| - Frequency of usage unclear | 1 (16.7) |
| - Platform seen as clear, intuitive, and self-explanatory | 5 (83.3) |
| **Integration** |  |
| - Good as a supplement | 6 (100) |
| - Therapy replacement possible | 1 (16.7) |
| - No replacement of therapy possible | 5 (83.3) |
| - Contents were not integrated | 5 (83.3) |
| - Could be more embedded in f2f | 2 (33.3) |
| - Viewed as part of therapy program (integrated) | 1 (16.7) |
| - Contents fit to therapy | 4 (66.7) |
| - Contents nothing new | 1 (16.7) |
| - Contents viewed as not matching | 1 (16.7) |
| - Different language version needed of some modules | 1 (16.7) |
| - Wish to work more independently | 1 (16.7) |
| - Positive outlook towards digitalization | 4 (66.7) |
| - Importance to adopt right attitude for the use of BT | 1 (16.7) |
| **Sequential BT** |  |
| - Wish not to use their own phone during stay / recommend extra device | 1 (16.7) |
| *Before the stay in hospital* |  |
| - Strange to receive module before admission | 3 (27.3) |
| - Wish for more information on symptoms before admission | 1 (16.7) |
| *After the stay in hospital* |  |
| - Would continue using after discharge | 2 (33.3) |
